# Hospital and Health News

**Published:** 1923-08

**Authors:** 


### HOSPITAL AND HEALTH NEWS.

### Alexandra Day.

A sum of £44,113 was collected in the streets of London for the hospitals on Alexandra Day.

### Congrès de Médecine.

The seventeenth meeting of the Congrès Français de Médecine will be held at Bordeaux from September 27th to 29th.

### Presentation to a Hospital Secretary.

Mr. Robert A. Mullan, who was Hon. Secretary to the Newry General Hospital from 1888 to 1922, has been presented with two portraits of himself, one of which is to hang in the board room.

### A Prince's Medal.

On the Prince of Wales's recent visit to the Wolverhampton and Staffordshire General Hospital, he was handed a cheque for £17,765 by a ten-year-old patient as the proceeds up to date of the hospital's appeal. In commemoration of his visit a special medal, designed by Mr. E. A. Cox, has been struck.

### Dangerous Drugs.

A handy little "Register for Recording All Purchases, Sales and Stock of Dangerous Drugs" is published at 3s. 6d. net by Messrs. John Wright, of Bristol. The Register is admirably arranged and tabulated, and is prefaced by extracts from the Regulations under the Dangerous Drugs Act.

### A Miners' Hospital.

The Miners' Hospital for Caerphilly, built at a cost of £30,000, has now been opened. The nucleus was a private house, which has been altered and added to extensively. The accommodation at present consists of 21 beds. Mr. T. Phillips is the Chairman, and Mr. Henry Richards, who has worked hard to carry the scheme through, is the Secretary.

### Insulin at Sheffield.

The Board of Sheffield Royal Infirmary has decided to appoint a clinical assistant to the physicians, who will devote himself particularly to the insulin treatment of diabetes. The gravest need at the moment is to increase the hospital accommodation of the city, which the free hospitals are now considering how best to do.

### Imperial Nurses' Club.

A sale is to take place in the autumn to raise money for building repairs at the Imperial Nurses' Club, 137 Ebury Street. Suggested gifts include "babies' needs, foreign and colonial wares, travellers' comforts, 'white elephants.'" All further details can be obtained from the hon. secretary, Miss Mayers, at Ebury Street.

### The Penny in the Pound.

Mr. Lamb, Secretary of the Sheffield Joint Hospital Council, has written a paper called "A Vision of Hospital Service in the Future." The paper, expressing his personal ideas, is written at a time when the success of the penny in the pound scheme has brought many inquiries to the council, which now hopes to tackle the grave problem of the scarcity of hospital beds in the city.

### Portrait of Sir Charles Bell.

A portrait of Sir Charles Bell, Surgeon to the Middlesex Hospital from 1814 to 1836, and founder of the Medical School in 1835, has been presented to the hospital by Mr. Alfred Webb-Johnson, the Dean of the Medical School, and has been placed in the Board Room. The portrait is the work of Mr. Dorofield Hardy, and has been modelled mainly on the bust in the Royal College of Surgeons.

### State Registered Nurses' Badge.

There will be considerable delay in issuing the State Registered Badge for Nurses to those who have made application, owing to the fact that a large number have omitted to give the particulars asked for. It is absolutely necessary that the full name of the nurse should be given, together with her registration number and the part of the Register on which she is registered, and postal order for 5s. 6d. in payment. Application for the badge should not be made until the Certificate of Registration has been received.

### For the Young Medical Practitioner.

The British Medical Association issue a "Handbook for Recently Qualified Medical Practitioners," containing "useful information and advice as to matters known to be often the subject of doubt or difficulty to entrants to the profession." The booklet, which is issued at the price of 2s. 6d. net, should prove invaluable to the young doctor.

### A North Staffordshire Triumph.

The North Staffordshire Infirmary has now extinguished the debt which as recently as October last amounted to £24,000. Mr. W. Stevenson, the House Governor, and Mrs. Harrison, the President, were loyally supported by every section of the community, and their achievement should be an inspiration to all placed where this hospital was a year ago.

### Nursing as a Profession.

The Association of Head Mistresses have published a pamphlet on "Nursing as a Profession." It is pointed out that there would seem to be some prejudice against nursing on the part of many girls and parents, and the object of this leaflet is to remove misconceptions and give accurate details about training, salaries and prospects, &c. It is certainly most desirable for the profession to attract girls of the educated classes, and this pamphlet should be distinctly useful.

### Massage An Ancient Art.

The first number of Volume IX. of "The Journal of the Chartered Society of Massage and Medical Gymnastics" which has a new and attractive cover, contains an interesting article on "Massage in Antiquity," by C. J. S. Thompson, with illustrations from old bas-reliefs. Massage was practised at a very early period in China, Japan and India, and the blind seem to have particularly cultivated the art. One of the most remarkable representations showing massage in operation is in a stone relief in the temple of Boro Budur in Central Java.

### "L'Infirmière Française."

We have received the first number of a new French monthly, *L'Infirmière Française*, issued under the patronage of the French Minister of Health and the personal direction of Professor Calmette, of the Pasteur Institute. The publication contains thoroughly practical articles by prominent medical men and well-known nurses, and will give particular attention to matters of public health. Intended for both male and female nurses, as well as for medical men engaged in their instruction, *L' Infirmière Française* (21 Rue Cassette, Paris) should meet with wide support.

### American Hospital Conference.

The American Hospital Association is holding its 25th Annual Conference at Milwaukee from October 29th to November 2nd. At this Conference Dr. Goldwater, who was present at the Sheffield Conference, will make his report. A larger amount of time is set apart this year for the exhibition of equipment and supplies, and most of the sessions of the Conference will be limited to an hour and a half. One session will be devoted to Hospital Standardisation, and another to various nursing problems. "Should General Hospitals Establish Departments of Therapy?" "Team Work Among Hospitals," and "Women's Work in Hospitals" are other subjects for discussion.

### Congress of Surgery.

The Prince of Wales opened the sixth Congress of the International Society of Surgery, which was inaugurated by a reception held at the University of London. The society has shown its sense of historical continuity by selecting as the subject of the year's medal the famous British surgeon John of Arderne, who flourished in the time of Edward III. For the most famous operation that he undertook he was awarded a fee of £100 and £200 a year for life and two suits of clothes, but it was one of the conditions that he should reside in the house of his patient until the latter was cured. Speakers at the Congress have included the Marquess Curzon and Mr. Neville Chamberlain.

### City Police War Memorial.

The Prince of Wales has opened the new Metropolitan and City Police War Memorial Hospital at Twickenham, on which £18,350 has been spent.

### Presentation to a Cottage Hospital.

A portrait of ex-Bailie William W. Hunter has been presented to the Denby Cottage Hospital, of which he was chairman from 1890 to 1922.

### Bequest to an Infirmary.

Leeds General Infirmary has received £26,208 as the proceeds of the residuary estate of the late Mrs. Henry Tiffany, whose death occurred a year ago.

### A Tuberculosis Hospital.

Grindon Hall, Sunderland's latest tuberculosis hospital for women and children, accommodates twenty-two adults and five children in four wards. Originally a private house, it stands in twelve acres mostly laid out as gardens.

### The Poor Women of Halifax.

In accordance with the will of the late Miss Ann Holt, a lady of Halifax, who died at Harrogate, a Rest Home for Poor Women of Halifax has been opened at Harrogate. The income of the trust is £3,000 and twelve women can be accommodated. Miss Thompson, formerly of Halifax Royal Infirmary, is matron.

### Adoption of Charing Cross Hospital.

The staff of the Hotel Cecil, which has adopted the Charing Cross Hospital, is hard at work raising sufficient to endow a bed there to be devoted to the use of any members who may fall ill. To maintain a bed requires £150 a year, and in twelve months the staff has raised £325 in the twenty-four collecting boxes distributed in the hotel.

### Charges for Medical Certificates.

The committee of Bedford County Hospital has decided to make the following charges for medical certificates:—For National Health Insurance and Friendly Society use, no charge; for newspaper insurance benefits, 5s.; for a report for Workmen's Compensation claims, 21s. The latter is said to be usually recovered from the insurance company concerned.

### Bibliography of Industrial Hygiene.

The First International Labour Office has published a Bibliography of Industrial Hygiene, the result of an examination of the most important periodicals of the medical press and other sources. It is to be issued as a separate quarterly publication in a single edition, and the headings under which the books and articles are grouped are given in French, English and German.

### Membership of the Institute of Hygiene.

Revised regulations governing the admission of candidates to membership of the Institute (M.I.H.) are now in force. Candidates holding certain alternative qualifications to the Diploma of the Institute in General Hygiene—the qualification hitherto accepted—are now admissible. Full details of the new regulations can be obtained from the Secretary at Devonshire Street, W. 1. Qualified members of the nursing profession are eligible for election.

### Pathology in the West.

A new pathological department has been opened at the Royal Devon and Exeter Hospital, which replaces the small room and mortuary where the work began. The number of examinations has risen steadily to a present average of 1,000 a year, and the usefulness of the addition will be increasingly recognised. The department has cost £1,800. To serve all the voluntary hospitals of the county area it is proposed that a pathologist should attend daily at a laboratory provided by the Dorset County Hospital at certain hours and receive a fee, possibly 2s., a head for all in or outpatients requiring his services. The scheme would also enable the general practitioners of the county to secure expert assistance.

### Tenement Houses in Kensington.

The Medical Officer of Health for Kensington states that the borough's 5,690 tenement houses are largely responsible for the high infantile death rate (82 per 1,000).

### City of Leicester Mental Hospital.

An X-ray apparatus has been added to the hospital during the year, a third assistant medical officer, capable of undertaking pathological work appointed, and certain rooms have been set apart for the use of the medical officers for psychotherapeutic purposes. The cost of the maintenance of a patient works out at just over a guinea a week, and the charge has been reduced to 17s. 6d. per week, owing to an accumulated balance from previous years.

### Glasgow Royal Maternity Hospital.

The work of this institution in all its branches continues to expand. In the last two years as many as 627 more cases were treated than in 1920. The total expenditure has fallen from £25,950 in 1921 to £22,500 in 1922, the cost of provisions showing the largest decrease. The almoner's department has been exceedingly active, and it was found necessary to appoint another worker. A visit was paid to the hospital from the British Medical Association last July and "the progress made since the Association last met in Glasgow (in 1888) was commented on in eulogistic terms, and in a publication of the Association reference is made to the high state of the hospital equipment."

### For the Guidance of Mothers.

Under the title of "Opening Doors," Dr. John Thomson has written a pamphlet for "the Mothers of Babies who are Long in Learning to Behave like other Children of Their Age." It is intended primarily for working-class mothers, and should be of real value to them. The advice, tactfully given, is thoroughly sound and practical, and should encourage a mother to feel a wholesome interest and pride in her child, even though it may be unusually dull or even definitely mentally defective. The little book is published by Messrs. Oliver and Boyd, of Edinburgh (1s. 6d. net), and may be specially recommended to child welfare workers and district nurses, and indeed to all who are brought into contact with the mothers of children whose mental powers are below the normal.

### The Royal Northern Hospital in Difficulties.

The Royal Northern Hospital is not an institution with a great historic past, nor can it claim the dimensions of a few of the large London hospitals. It is one of the newer hospitals which has had a rapid growth. Yet that growth is incommensurate with the growth of the population in North London for whose benefit it exists, and even more so with the expenditure necessary for its maintenance. Of the £85,000 which is required every year, under £3,000 is derived from investments. So each year the Board of Management are confronted with the task of securing £82,000. At the beginning of this year there was a deficiency of £36,300, which has grown to £50,000. This alarming situation has compelled the management to decide on the drastic and unwelcome course of closing some of the wards unless the sum of £20,000 can be raised by July 31st.

### The Pay of Medical Officers.

Dr. Keith, who was recently appointed Medical Officer of Health for St. Pancras, has withdrawn his acceptance of the post on the ground that London air is not good for his health. The Borough Council of St. Pancras is offering a salary of £1,000 per annum, which is considered inadequate by the British Medical Association. At the annual representative meeting of the British Medical Association at Portsmouth, the Council's scale of commencing salaries for Public Health Medical Officers was adopted. The scale for Medical Officers of Health ranges from £800, where the population is under 35,000 to £1,800 where the population exceeds 600,000. The Council's scheme for co-operation between the Association and the Society of Medical Officers of Health was likewise adopted. Consideration of the recommendation that a percentage of payments made for hospital benefits should be passed into a fund at the disposal of the honorary medical staff of the hospital, was postponed until next year.

